# Therapeutic Effects of Traditional Chinese Medicine for Patients With Coronary Heart Disease After Treatment of Revascularization: A Prospective Cohort Study in the Northern of China

**DOI:** 10.3389/fcvm.2021.743262

**Published:** 2021-11-17

**Authors:** Chunxiao Lv, Zuozhang Cheng, Hao Yu, Huiqing Du, Yingqiang Zhao, Yujie Liu, Junhua Zhang, Sheng Gao, Ruifeng Liu, Yuhong Huang

**Affiliations:** ^1^Department of Clinical Pharmacology, Second Affiliated Hospital of Tianjin University of Traditional Chinese Medicine, Tianjin, China; ^2^Department of Geriatrics, Changle People's Hospital, Weifang, China; ^3^Department of Statistics, Nanjing Medical University, Nanjing, China; ^4^Department of Emergency, Qidong Hospital of Traditional Chinese Medicine, Nantong, China; ^5^Department of Cardiology, Second Affiliated Hospital of Tianjin University of Traditional Chinese Medicine, Tianjin, China; ^6^Department of Cardiology, Tianjin Chest Hospital, Tianjin, China; ^7^Evidence-Based Medicine Centre, Research Institute of Traditional Chinese Medicine, Tianjin University of Traditional Chinese Medicine, Tianjin, China; ^8^Department of General Practice, Tianjin Hospital of Intergrated Traditional Chinese and Western Medicine Nankai Hospital, Tianjin, China; ^9^Department of Cardiology, Traditional Chinese Medicine Hospital of Beichen District, Tianjin, China

**Keywords:** coronary heart disease (CHD), revascularization, Traditional Chinese Medicine (TCM), prospective cohort study, therapeutic effects

## Abstract

**Aim:** To investigate the compliance and the outcome of Traditional Chinese Medicine (TCM) in patients with coronary heart disease (CHD) after treatment of revascularization.

**Methods:** In this prospective cohort study, the non-exposure group (NEG), low-exposure group (LEG), and high-exposure group (HEG) were divided after 2 years follow-up. The primary outcome was a composite of death from cardiovascular causes, non-lethal myocardial infarction, heart transplantation, or stroke. Time-to-event data were evaluated by using the Cox regression analysis with hazard ratios (HRs) and 95% CIs. Then, the two-sided *p*-values were calculated by using the Cox models. In order to indicate the therapeutic effects of TCM on the CHD after revascularization, the survival analysis and the nested case–control study were conducted separately.

**Results:** There were 1,003 patients with CHD enrolled, 356 patients (35.49%) did not choose the TCM, 379 patients (37.79%) used the TCM seldom, and only 268 patients (26.72%) used TCM regularly. A total of 653 patients with revascularization participated in the prospective cohort study. Over the duration of the trial, the primary endpoints occurred in 12 (4.35%), 11 (4.80%), and 2 (1.35%) patients in the NEG, LEG, and HEG, while the secondary endpoints occurred in 84 (30.43%), 57 (24.89%), and 15 (10.14%) patients in the NEG, LEG, and HEG, respectively. The occurrence time of secondary endpoint events in HEG was significantly postponed (*p* < 0.001) compared with the other cohorts. The Cox regression analysis indicated that the HRs in the primary endpoints, the secondary endpoint events, the major adverse cardiac and cerebrovascular events (MACCE), and the composite endpoint events for HEG were all around 0.3 (*p* < 0.05) and HRs for LEG were all around 0.8. The results of the nested case–control study showed that the TCM exposure was significantly different between the cases and controls in the secondary endpoints (*p* < 0.05), while no significant difference in the primary endpoints (*p* > 0.05), but the percentage of HEG in the cases was extremely lower than the controls.

**Conclusion:** The HEG-TCM may improve the outcomes of the patients with CHD after treatment of revascularization.

**Registration:** http://www.chictr.org.cn. Unique identifier: ChiCTR-OOC-17012995.

## Introduction

Currently, cardiovascular disease (CVD) is the leading cause of premature morbidity and mortality in the world (1–6), of which coronary heart disease (CHD) is the most prevalent and accounts for one-third to one-half of the CVD cases (7). In 2017, an estimated 17.8 million people died of CVD, almost 31% of all the deaths globally (1, 8) and CHD represented 64% of all the CVD-related deaths (9, 10). In the United States, the prevalence of CHD is more than 12 million and ~2 million people suffer from CHD in the United Kingdom (11). In China, there are over 11 million people diagnosed of CHD which is expected to increase steadily with further economic development in the next few decades (12, 13).

Coronary heart disease, as a chronic condition, caused the further cardiac damage and death (14, 15). Patients with CHD might be individual differences in pathogenesis, disease progression, and genetic background, which need combination of several conventional Western medicines to relief symptoms and the risk of adverse effects would increase. Also, the patients with CHD require long-term medical care to improve their quality of life and reduce morbidity and mortality (16). Traditional Chinese medicine (TCM) is of natural origin, without many adverse effects (17), which would be a relatively safe treatment. TCM with multitargets, multipathways, and individual regimen is significant complementary to the Western medicine and is an effectively alternative therapy for the patients, therefore commonly used in CHD treatments in China and other Asian countries (18). Although it is always reported that many TCM have been widely used to treat CHD in clinical practice, there are still lack more information on practice patterns and therapeutic effects of TCM treatment, especially for disease management and endpoints (19, 20).

Accordingly, the TCM treatment accepted by the patients with CHD was investigated and a prospective cohort study was carried out to learn about the compliance and prevalence of endpoint events in the patients with CHD after treatment of revascularization. Then, it presents a valuable individual therapy strategy with implications for disease management and endpoints for the patients with CHD.

## Methods

### Study Design

From October 2017 to October 2019, a prospective cohort study was conducted in three representative hospitals of Tianjin. They were Tianjin Chest Hospital representing Western Medical Hospital, the Second Affiliated Hospital of Tianjin University of TCM, and the TCM Hospital of Beichen District representing TCM hospital to get the situation about the TCM treatment accepted by the patients with CHD and evaluate the therapeutic effects of TCM on the patients with CHD after treatment of revascularization. The medicines used, symptoms, and clinical endpoint events of the participants were collected in the baseline and every 3 months without interfering with the treatment of the patients.

This study was registered on the China Clinical Trial Registry with number ChiCTR-OOC-17012995. Additionally, this study was conducted in accordance with the recommendations of Ethics Committees of the Second Affiliated Hospital of Tianjin University of TCM and the protocol was approved by the Ethics Committees with registration number 2017-046-01. All the subjects gave a written informed consent form (ICF) in accordance with the Declaration of Helsinki.

### Participants

The patients who were diagnosed as CHD [the CHD diagnostic criteria were: (1) the lumen diameter of at least one main branch was narrowed by more than 50% which were confirmed by coronary angiography or coronary CT angiography and (2) there was clear evidence for myocardial infarction in the previous or at that time including ST-segment elevation or non-ST-segment elevation] for more than 5 years would be screened. The exclusion criteria were as follows: (1) Patients with severe cardiopulmonary insufficiency and severe primary organ diseases before the recruitment such as tumor; (2) Mental patients; (3) Participants joined in other interventional clinical research; (4) Patients with poor compliance and did not cooperate well; and (5) Those who had no crucial data such as refusing to give feedback to the visit and failing to answer the call for many times. All the excluded cases should be recorded and the case report forms should be kept for future reviewing, but without statistical analysis. The patients who experienced revascularization including percutaneous coronary intervention (PCI), coronary artery bypass grafting (CABG), or both would be enrolled into the prospective cohort study.

### Traditional Chinese Medicine Cohorts

Over the duration of the clinical trial, the participants were divided into three cohorts, the non-exposure group (NEG), low-exposure group (LEG), and high-exposure group (HEG), according to the administration amount and duration time of TCM treatment (including proprietary Chinese medicine and decoction). In the NEG, participants did not use any TCM during the visiting periods; in LEG, participants did not follow the direction of the physicians or irregularly used TCM during the visiting periods or they did not use TCM enough, which was defined as the period of continuous using TCM < 2 months or intermittent using TCM (recorded the cumulative time) < 4 months; in HEG, participants continuously followed the prescribed regimens with a long-term using of TCM, which was defined as the period of regular using TCM ≥ 2 months or intermittent using TCM ≥ 4 months.

### Endpoint Events

In this study, the primary endpoint was a composite of death from cardiovascular causes, non-lethal myocardial infarction, heart transplantation, or stroke. The secondary endpoint was repeat revascularization. The total of primary and secondary endpoint was identified as the cardiovascular composite endpoint events. The major adverse cardiac and cerebrovascular events (MACCE) included primary endpoint events, secondary endpoint events, and all-cause mortality.

### Data Collection, Management, and Quality Control

All the research-related information was inputted separately by the two researchers who were trained rigorously. The consistency was checked automatically and the queries were sent and responded when the differences were founded during the checking. All the information of the participants would be stored securely in locked file with limited access.

### Statistical Analysis

Continuous variables were expressed as mean ± SD and categorical variables were expressed as counts and percentages. The SPSS software version 26.0 (USA) was used for all the statistical analysis. A two-sided *p* ≤ 0.05 was considered as statistical significant.

Baseline characteristics in different cohorts were tested by using the chi-squared test or the Fisher's exact test for categorical variables (e.g., gender) and the ANOVA for continuous variables (e.g., age).

The incidence of endpoint events and correlation intensity [relative risk/hazard ratio (RR/HR)] was calculated and the statistical inference was carried out by survival analysis. Moreover, the cumulative survival rate and death trend were described by the Kaplan–Meier (KM) curve where long-rank test was used to evaluate the survival rate between cohorts. Then, the multivariate Cox regression models with HRs and 95% CI were used to analyze the multivariate factors in CHD. If HRs ≈ 1 indicating that the factors do not affect CHD; if HRs < 1 indicating a protective factor; if HRs > 1 indicating a dangerous factor.

## Results

### Participants

**Figure 1:**
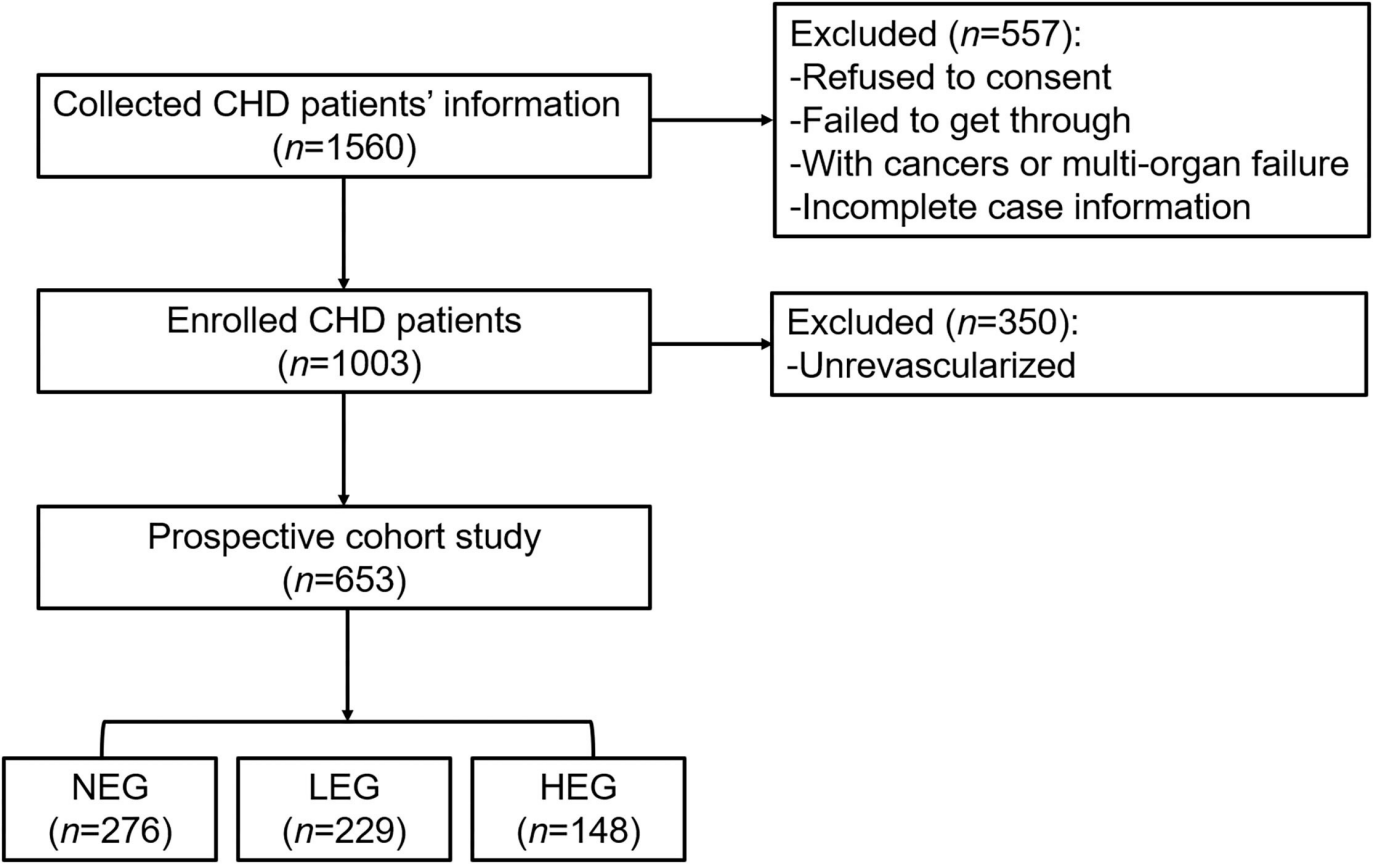
The flowchart of the study. TCM, Traditional Chinese Medicine; NEG, non-exposure group; LEG, low-exposure group; HEG, high-exposure group.

The study flow was shown in Figure 1. A total of 1,560 patients with CHD were screened from three hospitals and 557 (35.71%) patients did not fulfill the inclusion criteria, so there were 1,003 (64.29%) patients enrolled. Except the 350 (34.90%) patients without the revascularization therapy, 653 (65.10%) participants participated in the prospective cohort study. According to the TCM treatment, there were 276 (42.27%), 229 (35.07%), and 148 (22.66%) participants in the NEG, LEG, and HEG, respectively. At the last assessment, there were 540 (82.70%) revascularization patients who kept on the 2 years follow-up or in whom the aforementioned endpoint occurred. 113 (17.30%) revascularization patients without endpoint events did not last for 2 years and their data were censored at the last contact.

### Baseline Characteristics of the Patients

A total of 1,003 patients with CHD were investigated for the baseline characteristics (Supplementary Table 1). The average age was 66.68 ± 9.16 years and the course of CHD was 9.72 ± 5.71 years. Of these patients, 568 (56.63%) patients were male, 228 (22.73%) patients drunk alcohol, 467 (46.56%) patients were smoker, and 138 (13.76%) patients had the family history of CHD. The most combined disease was hypertension occupying 78.96% (792 patients) and the least one was the atrial fibrillation (AF), which accounted for 8.27% (83 patients). Most of the patients accepted three or more drugs simultaneously including herbal medicine. A total of 829 (82.65%) patients were administrated with the lipid-lowering agents (the most) and 36 (3.59%) patients were administrated with anticoagulants (the least). The sequence of medicines that the patients preferred to was lipid-lowering agents > nitrates > TCM > β-blockers > dual antiplatelet therapy (clopidogrel + aspirin) > angiotensin-converting enzyme inhibitor (ACEI) and angiotensin receptor blocker (ARB) > calcium channel blockers > anticoagulants. There were 639 (63.71%) patients expressing that they would like to try the TCM treatment for the CHD, while 647 (64.51%) patients took TCM in fact. Of 1,003 patients with CHD, 356 (35.49%) patients did not choose the TCM, 379 (37.79%) patients used the TCM seldom, and only 268 (26.72%) patients used TCM regularly. Most patients were familiar with the TCM, but they were not liable to use it regularly attributed to the unhappy taste, inconvenience, or chronic effect, although TCM with multitargets and multicomponents would be a significant complementary therapy.

The baseline characteristics of the cohort participants are shown in Table 1. There were 653 patients enrolled, 276 (42.27%), 229 (35.07%), and 148 (22.66%) participants in the NEG, LEG, and HEG, respectively. These participants were from 42 to 93 years old with the average age of 66.55 ± 8.93 years. The participants suffered from CHD for 5–43 years without significant difference between the three cohorts. Also, there was not any difference in combination diseases, drugs administration, and smoking and drinking alcohol hobby between three TCM cohorts, except of cerebral infarction, taking dual antiplatelet therapy (clopidogrel + aspirin), and nitrates drugs.

**Table 1:** Baseline characteristics of the study participants in the cohort study (*n* = 653).

**Characteristics**	**NEG (%)**	**LEG (%)**	**HEG (%)**	***χ2/F***	***P***
Age (years)	65.51 ± 8.74	67.65 ± 8.85	66.76 ± 9.22	3.70	0.0254
Age group				10.20	0.2541
41–50 years	11 (3.99)	6 (2.62)	6 (4.05)		
51–60 years	67 (24.28)	43 (18.78)	28 (18.92)		
61–70 years	116 (42.03)	98 (40.17)	67 (45.27)		
71–80 years	73 (26.45)	71 (31.00)	36 (24.32)		
81–90 years	9 (3.26)	17 (7.42)	11 (7.43)		
Gender				5.01	0.0817
Male	184 (66.67)	132 (57.64)	87 (58.78)		
Female	92 (33.33)	97 (42.46)	61 (41.22)		
CHD duration (years)	9.38 ± 4.71	9.92 ± 6.41	9.95 ± 5.43	0.79	0.4556
Duration group				7.24	0.5160
5–10 years	169 (61.23)	138 (60.26)	86 (58.11)		
10–15 years	65 (23.55)	60 (26.20)	40 (27.03)		
15–20 years	24 (8.70)	11 (4.80)	11 (7.43)		
20–30 years	16 (5.80)	13 (5.68)	8 (5.41)		
≥30 years	2 (0.72)	7 (3.06)	3 (2.03)		
Drinking alcohol				5.35	0.0687
No	205 (74.28)	185 (80.79)	105 (70.95)		
Yes	71 (25.72)	44 (19.21)	43 (29.05)		
Smoking history				5.80	0.0550
No	124 (44.9)	127 (55.5)	77 (52.0)		
Yes	152 (55.1)	102 (44.5)	71 (48.0)		
Family history of CHD				4.81	0.0903
No	245 (88.77)	198 (86.46)	120 (81.08)		
Yes	31 (11.23)	31 (13.54)	28 (18.92)		
Heart rhythm				3.69	0.4501
Regular	55 (19.93)	53 (23.14)	37 (25.00)		
Arrhythmia (No AF)	203 (73.55)	154 (67.25)	100 (67.57)		
AF	18 (6.52)	22 (9.61)	11 (7.43)		
NYHA				5.58	0.0625
I	23 (8.33)	8 (3.49)	2 (1.35)		
II	215 (77.90)	185 (80.79)	120 (81.08)		
III	30 (10.87)	27 (11.79)	24 (16.89)		
IV	8 (2.90)	9 (3.93)	1 (0.68)		
AMI				0.62	0.7345
No	183 (66.30)	159 (69.30)	98 (66.22)		
Yes	93 (33.69)	70 (30.70)	50 (33.79)		
Cerebral infarction				8.29	0.0159
No	233 (84.42)	179 (78.07)	108 (72.97)		
Yes	43 (15.58)	50 (21.93)	40 (27.03)		
AMI and cerebral infarction				2.01	0.3663
No	261 (94.57)	211 (92.14)	135 (91.22)		
Yes	15 (5.43)	18 (7.86)	13 (8.78)		
Diabetes				0.59	0.7450
No	161 (58.33)	137 (59.83)	92 (62.16)		
Yes	115 (41.67)	91 (40.17)	56 (37.84)		
Hyperlipidemia				0.61	0.7382
No	242 (87.68)	196 (85.59)	130 (87.84)		
Yes	34 (12.42)	33 (14.41)	18 (12.16)		
Hypertension				5.76	0.0562
No	70 (25.36)	38 (16.59)	31 (20.95)		
Yes	206 (74.64)	191 (83.41)	117 (79.05)		
Combined diseases				4.06	0.1312
0	34 (12.42)	18 (7.86)	15 (10.14)		
1	94 (34.06)	71 (31.00)	51 (34.46)		
2	113 (40.94)	102 (44.54)	61 (41.22)		
3	32 (11.59)	36 (15.72)	18 (12.16)		
4	3 (1.09)	2 (0.87)	3 (2.03)		
Dual-antiplatelet (Clopidogrel + Aspirin)				20.55	0.0000
No	84 (30.43)	94 (41.05)	78 (52.70)		
Yes	192 (69.57)	135 (58.95)	70 (47.30)		
Anticoagulants				1.90	0.3863
No	268 (97.10)	217 (94.76)	141 (95.27)		
Yes	8 (2.90)	12 (5.24)	7 (4.73)		
Lipid-lowering agents				0.25	0.8816
No	43 (15.58)	33 (14.41)	24 (16.22)		
Yes	233 (84.42)	196 (85.59)	124 (83.78)		
Calcium channel blockers				4.60	0.1001
No	190 (68.84)	138 (60.26)	91 (61.49)		
Yes	86 (31.16)	91 (39.74)	57 (38.51)		
β-blockers				2.49	0.3031
No	86 (31.16)	75 (32.89)	57 (38.51)		
Yes	190 (68.84)	153 (67.11)	91 (61.49)		
ACEI & ARB				1.99	0.3693
No	145 (52.54)	133 (58.08)	86 (58.11)		
Yes	131 (47.46)	96 (41.92)	62 (41.89)		
Nitrates				6.40	0.0407
No	99 (35.87)	59 (25.76)	51 (34.46)		
Yes	177 (64.13)	170 (74.24)	97 (65.54)		

*TCM, Traditional Chinese Medicine; NEG, non-exposure group; LEG, low-exposure group; HEG, high-exposure group; CHD, coronary heart disease; AF, atrial fibrillation; NYHA, New York Heart Association; AMI, acute myocardial infarction; ACEI, angiotensin-converting enzyme inhibitor; ARB, angiotensin receptor blocker*.

### Effect of TCM on Cumulative Endpoint Events

The incidence of endpoint events is shown in Table 2. The cumulative incidence of endpoint events was increased with time. Through the 2 years follow-up, the primary endpoints occurred in 12 (4.35%), 11 (4.80%), and 2 (1.35%) revascularization patients in the NEG, LEG, and HEG, respectively, while the secondary endpoints occurred in 84 (30.43%), 57 (24.89%), and 15 (10.14%) revascularization patients in the NEG, LEG, and HEG, respectively. The total of primary and secondary endpoints as 96 (34.78%), 68 (29.69%), and 17 (11.49%) in the NEG, LEG, and HEG, respectively. Moreover, the MACCE events took place in 97 (35.14%), 72 (31.44%), and 19 (12.84%) revascularization patients in the NEG, LEG, and HEG (*p* < 0.001).

**Table 2:** The incidence of the cumulative endpoint events.

	**Group**	**6 months period**	**12 months period/Total**	**18 months period/Total**	**24 months period/Total**
	**(participants)**	**(%)**	**(%)**	**(%)**	**(%)**
The primary endpoint events	NEG (276)	4 (1.45)	6/10 (3.62)	1/11 (3.99)	1/12 (4.35)
	LEG (229)	4 (1.75)	3/7 (3.06)	1/8 (3.49)	3/11 (4.80)
	HEG (148)	0 (0)	0/0 (0)	2/2 (1.35)	0/2 (1.35)
The secondary endpoint events	NEG (276)	34 (12.32)	23/57 (20.65)	24/81 (29.35)	3/84 (30.43)
	LEG (229)	16 (6.99)	18/34 (14.85)	18/52 (22.71)	5/57 (24.89)
	HEG (148)	5 (3.38)	5/10 (6.76)	0/10 (6.76)	5/15 (10.14)
MACCE	NEG (276)	38 (13.77)	30/68 (24.64)	25/93 (33.70)	4/97 (35.14)
	LEG (229)	20 (8.73)	22/42 (18.34)	20/62 (27.07)	10/72 (31.44)
	HEG (148)	6 (4.05)	6/12 (8.11)	2/14 (9.46)	5/19 (12.84)
The composite endpoint events	NEG (276)	38 (13.77)	29/67 (24.28)	25/92 (33.33)	4/96 (34.78)
	LEG (229)	20 (8.73)	20/40 (17.47)	20/60 (26.20)	8/68 (29.69)
	HEG (148)	5 (3.38)	5/10 (6.76)	2/12 (8.11)	5/17 (11.49)

*TCM, Traditional Chinese Medicine; NEG, non-exposure group; LEG, low-exposure group; HEG, high-exposure group*.

### Effect of TCM on Cumulative Survival Rate

**Figure 2:**
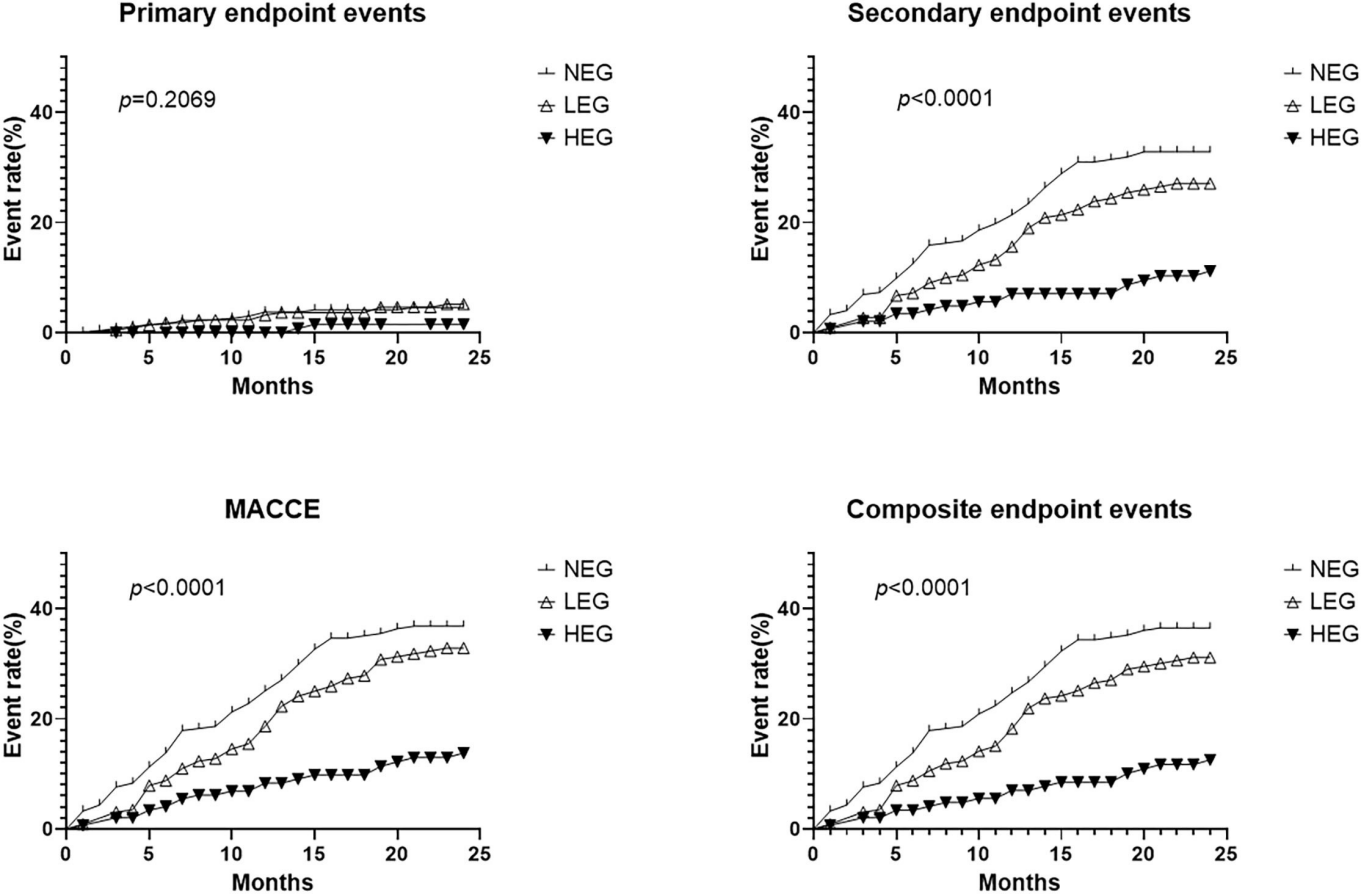
The Kaplan–Meier survival curves of the primary endpoint events, secondary endpoint events, the major adverse cardiac and cardiovascular events (MACCE), and the composite endpoint events. The long-rank test was used to compare the survival rate between different TCM cohorts. *p*-value was 0.2069 for primary endpoint events and *p*-values were <0.001 for secondary endpoint events, the MACCE, and the composite endpoint events, respectively. TCM, Traditional Chinese Medicine; NEG, non-exposure group; LEG, low-exposure group; HEG, high-exposure group.

From the KM curves (Figure 2), the occurrence time of secondary endpoint events, the MACCE, and the composite endpoint events in HEG were significantly postponed (*p* < 0.001) compared with the other cohorts. Additionally, the difference between the NEG and LEG was not significant and the trends were similar in both of the cohorts. The risk of primary endpoint events did not show and significant difference (*p* = 0.2069) between the three cohorts. In addition, the incidence of primary endpoints was much lower than the secondary endpoints (Table 2). Thus, HEG-TCM treatment could probably increase the cumulative survival rate of the patients with CHD after revascularization.

### Association Between Risk Variables and Endpoints

The COX regression models were used to analyze the association between the risk variables and endpoints. Firstly, using the univariate Cox models to identify the significant variables on endpoints (*p* < 0.05). Then, these significant variables along with the variables (cerebral infarction, nitrates, smoking history, and cardiac function), which were reported to impact the CHD significantly (21–23), were considered as the risk variables and introduced into the multivariate Cox regression models (Table 3). For primary endpoint events, the HEG-TCM might be a protective factor (HR = 0.3, <1), the clopidogrel + aspirin possibly was a dangerous factor (HR = 2.97, >1), while the other variables did not affect CHD seriously (HR ≈ 1). For secondary endpoint events, it is indicated that the HEG-TCM was also a protective factor and clopidogrel + aspirin and the New York Heart Association Class IV (NYHA-IV) were risk factors. Furthermore, LEG-TCM possibly was a protective factor for the primary and secondary endpoints, the MACCE, and the composite endpoint events (HR = 0.8, <1) in the patients with CHD with revascularization.

**Table 3:** The relationship between the multivariate factors and CHD analyzed by the Cox regression models.

	**Factors**		**HR (95% CI)**	***P***
The primary endpoint events	LEG	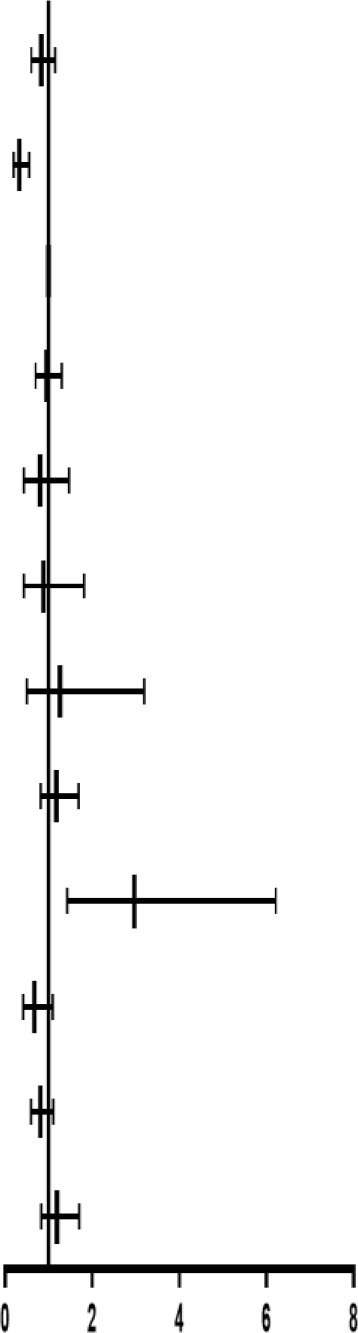	0.831 (0.603–1.146)	0.2587
	HEG		0.331 (0.195–0.560)	< .0001
	Age		0.996 (0.978–1.014)	0.6507
	Smoking-history		0.955 (0.701–1.302)	0.7726
	NYHA-II		0.800 (0.436–1.467)	0.4709
	NYHA-III		0.882 (0.430–1.812)	0.7330
	NYHA-IV		1.260 (0.497–3.193)	0.6261
	Cerebral-infarction		1.174 (0.815–1.690)	0.3896
	Dual-antiplatelet		2.970 (1.420–6.213)	0.0038
	Lipid-lowering agents		0.674 (0.414–1.097)	0.1120
	ACEI&ARB		0.812 (0.597–1.105)	0.1849
	Nitrates		1.189 (0.829–1.705)	0.3467
The secondary endpoint events	LEG	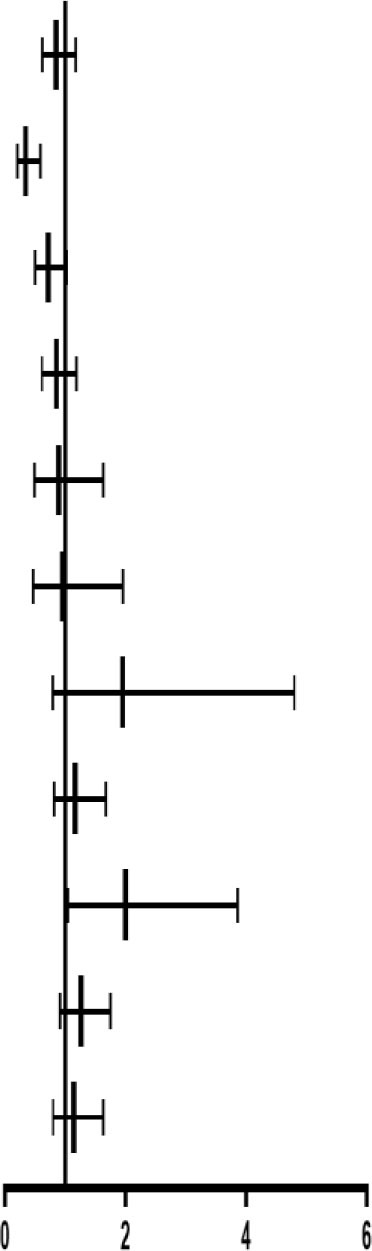	0.850 (0.616–1.172)	0.3208
	HEG		0.344 (0.204–0.583)	< .0001
	Gender, female		0.715 (0.502–1.012)	0.0642
	Smoking-history		0.854 (0.614–1.185)	0.3455
	NYHA-II		0.891 (0.486–1.633)	0.7096
	NYHA-III		0.952 (0.464–1.955)	0.8940
	NYHA-IV		1.950 (0.792–4.799)	0.1461
	Cerebral-infarction		1.165 (0.811–1.674)	0.4093
	Dual-antiplatelet		2.002 (1.039–3.858)	0.0381
	β-blockers		1.262 (0.910–1.752)	0.1637
	Nitrates		1.140 (0.799–1.626)	0.4712
MACCE	LEG	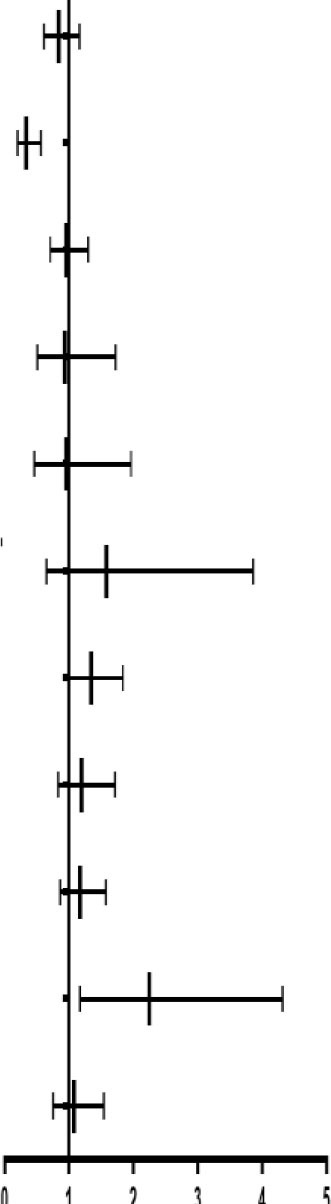	0.842 (0.611–1.162)	0.2955
	HEG		0.335 (0.198–0.567)	< .0001
	Smoking-history		0.960 (0.710–1.299)	0.7886
	NYHA-II		0.935 (0.508–1.724)	0.8306
	NYHA-III		0.954 (0.463–1.965)	0.8990
	NYHA-IV		1.583 (0.649–3.860)	0.3129
	AMI		1.345 (0.985–1.837)	0.0620
	Cerebral-infarction		1.193 (0.830–1.714)	0.3401
	Diabetes		1.169 (0.867–1.576)	0.3048
	Dual-antiplatelet		2.247 (1.169–4.316)	0.0151
	Nitrates		1.075 (0.750–1.540)	0.6935
The composite endpoint events	LEG	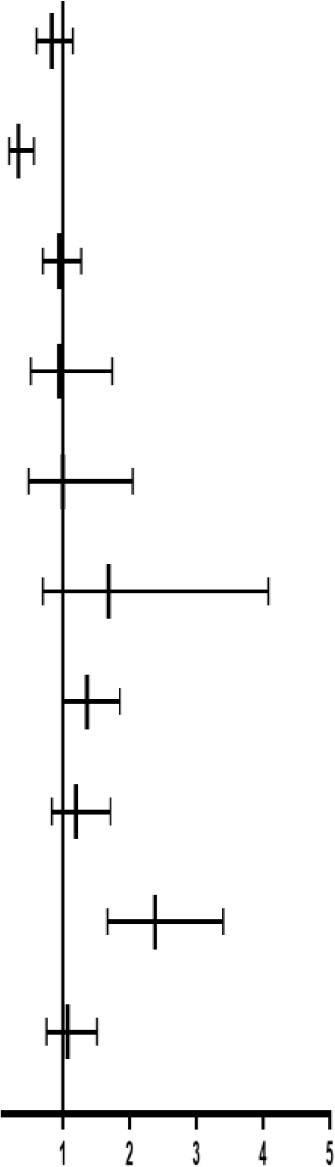	0.834 (0.605–1.149)	0.2657
	HEG		0.333 (0.197–0.563)	0.0000
	Smoking-history		0.941 (0.697–1.272)	0.6937
	NYHA-II		0.944 (0.513–1.737)	0.8524
	NYHA-III		0.998 (0.487–2.046)	0.9957
	NYHA-IV		1.685 (0.696–4.082)	0.2477
	AMI		1.357 (0.994–1.853)	0.0544
	Cerebral-infarction		1.191 (0.829–1.710)	0.3439
	Dual-antiplatelet		2.381 (1.666–3.404)	0.0000
	Nitrates		1.067 (0.756–1.507)	0.7106

*TCM, Traditional Chinese Medicine; NEG, non-exposure group; LEG, low-exposure group; HEG, high-exposure group; NYHA, New York Heart Association; ACEI, angiotensin-converting enzyme inhibitor; ARB, angiotensin receptor blocker; AMI, acute myocardial infarction*.

*Dual-antiplatelet therapy is for clopidogrel + aspirin*.

### Nested Case–Control Study for the Primary Endpoints and Secondary Endpoints

Case was defined as participant with primary endpoints or secondary endpoints, respectively. Control was defined as participant in whom any aforementioned endpoints did not occur during the study. Moreover, cases and controls were matched on sex and age in the window of 3 years (1:1 matching), which were reported to significantly affect the occurrence of CHD endpoints (18) by using propensity score-matched analysis. When the 25 cases were compared with 628 controls in the primary endpoints, the 2 (8.00%) patients with HEG were in the cases and 146 (23.25%) patients with HEG were in the controls. When the 156 cases were compared with 497 controls in the secondary endpoints, these 15 cases (9.62%) and 133 controls (26.76%) were in the HEG. Also, the results of the sex and age matching cases were almost the same. For the secondary endpoints, the cases were 15 (9.26%) in the HEG, 57 (36.54%) in LEG, and 84 (53.85%) in the NEG and the controls were 133 (26.76) in the HEG, 172 (34.61) in LEG, and 192 (38.63) in the NEG; the TCM exposure were significantly different between the cases and controls (*p* < 0.05), while no significant difference for the primary endpoints (*p* > 0.05) (Table 4). The nested case–control results furthermore indicated that regular TCM treatment would a protective factor in the patient with CHD with revascularization.

**Table 4:** The results from the nested case–control study.

	**Endpoints**		**NEG (%)**	**LEG (%)**	**HEG (%)**	**Total**	***P***
Non-matched	Primary endpoints	Cases	12 (48.00)	11 (44.00)	2 (8.00)	25	0.170
		Controls	264 (42.04)	218 (34.71)	146 (23.25)	628	
	Secondary endpoints	Cases	84 (53.85)	57 (36.54)	15 (9.62)	156	0.000
		Controls	192 (38.63)	172 (34.61)	133 (26.76)	497	
Matched on sex and age in 3 years	Primary endpoints	Cases	12 (48.00)	11 (44.00)	2 (8.00)	25	0.212
		Controls	9 (36.00)	9 (36.00)	7 (28.00)	25	
	Secondary endpoints	Cases	84 (53.85)	57 (36.54)	15 (9.62)	156	0.002
		Controls	65 (41.67)	53 (33.97)	38 (24.36)	156	

*TCM, Traditional Chinese Medicine; NEG, non-exposure group; LEG, low-exposure group; HEG, high-exposure group*.

### Frequency of TCM Used in Cohort Study

**Figure 3:**
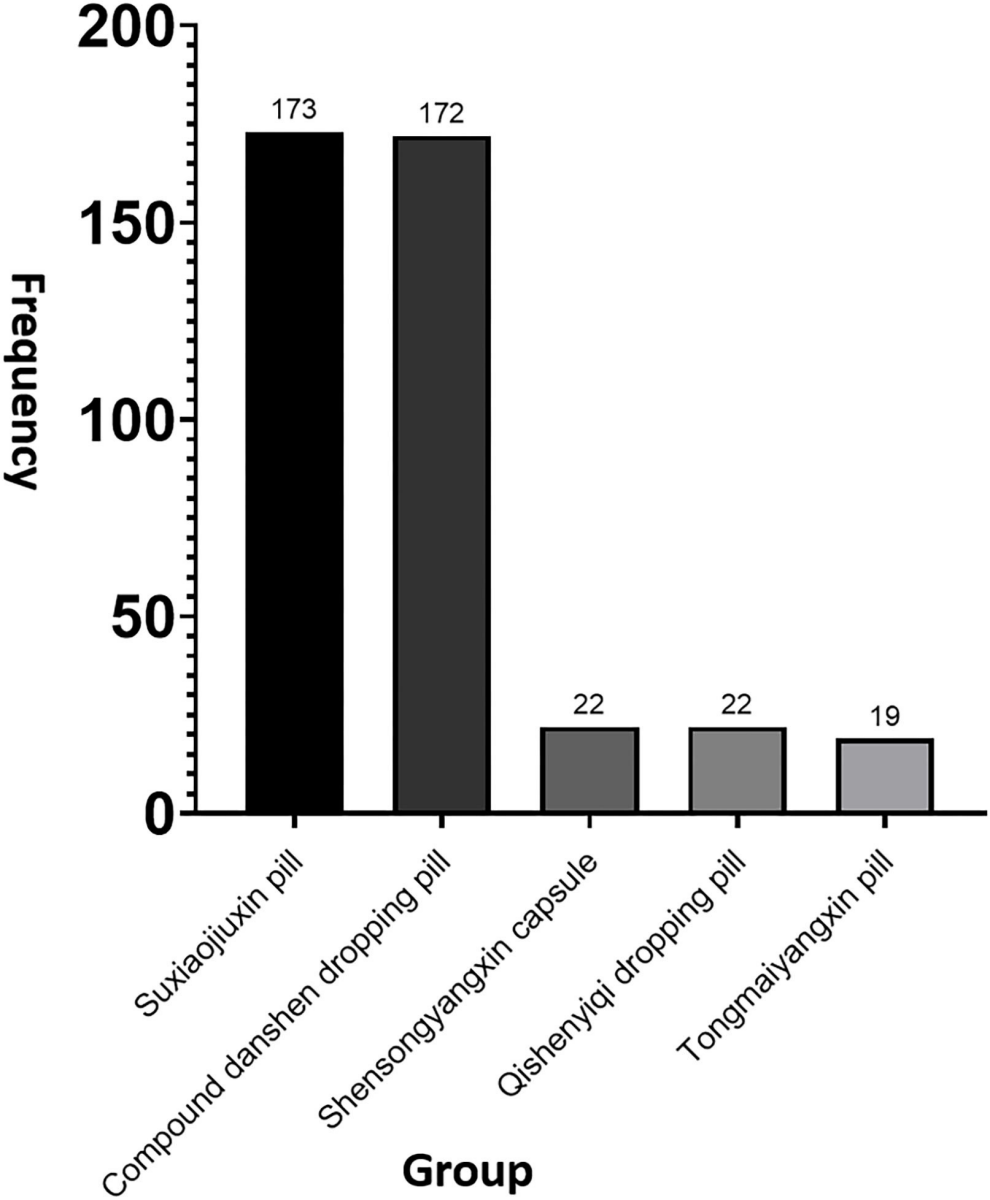
The top five used the TCM in cohort study. Totally, there were 173 patients using Suxiaojiuxin pill, 172 patients using compound Danshen dropping pill, 22 patients using Shensongyangxin capsule and Qishen Yiqi dropping pill, and 19 patients using Tongmai Yangxin pill.

In the prospective cohort study, the participants took TCM in LEG and HEG cohort. It showed the top five used frequency TCM, which were prescribed individually according to the disease syndrome by TCM physicians (Figure 3). There were 136 and 37 patients by using Suxiaojiuxin pill in the LEG and HEG, while for compound Danshen dropping pill, there were 119 and 53 patients in the LEG and HEG, respectively.

## Discussion

Coronary heart disease has become a global health problem increasingly (15). In the Donghan dynasty (25–220 AD), the pathogenesis and treatment of CHD were recorded in the Treatise on Febrile and Miscellaneous Disease whose author was Zhang Zhongjing honored as “Medical Sage.” So far, the pathogenesis has been accepted and the treatment has been administrated widely in the clinical practice. Nevertheless, the practice pattern (such as were patients like the TCM?, how was the compliance of patients taking TCM?, and what is the TCM regimen?) was not clear and there is less evidence to prove its therapeutic effects. In this study, we did a pilot study to investigate the practice patterns and to present the effectiveness of TCM on CHD with revascularization. A total of 1,003 patients diagnosed as CHD more than 5 years ago were investigated for the basic characteristics. The risk of stroke and myocardial infarction increased over time and became the main cause of death at 5 years (21), so that the patients with 5-years duration of CHD were considered. The demographic information, some risk factors (such as aging, hypertension, hyperlipidemia, diabetes, and so on), and the TCM treatment were recorded. The hypertension was the top one in all the combined diseases. Some references have demonstrated that incidence of CHD was significantly associated with hypertension, which was the largest risk factor and the prevention of hypertension was the fundamental and essential strategy to prevent CHD (23–25). Moreover, most of the patients preferred lipid-lowering agents, which were consistence with some other clinical results that reduction of the plasma lipid levels could prevent from CHD events (26). The prescription of the TCM is personalized according to the development of the syndrome, i.e., the individual treatment (27). The special taste and inconvenience of the TCM limit are its popularity. The favor of the patient is a key role to decide if he keeps on taking the TCM, although it can work in the disease cure. Thus, it is necessary to comprehensively evaluate the effectiveness of the TCM on CHD and guide patients to use TCM properly.

The cohort study with long time and large samples can follow the individualized diagnosis and dynamic changes of treatment of TCM (28). Moreover, the bias of the data would be smaller in this study because the medicine exposure and endpoints could be directly obtained (28). Thus, this study was a prospective cohort study that only the patients who experienced the revascularization would be enrolled because they would be easy to occur the endpoints and might be sensitive to treatment of the TCM (29) due to the large ischemic myocardial areas of the patients. The results of this study showed taking TCM regularly with a longer time that significantly decreased the incidence of repeat revascularization and delayed the occurrence of primary endpoint events such as death from cardiovascular causes, non-lethal myocardial infarction, heart transplantation, and stroke in the patient with CHD revascularization. Using the Cox regression models, HEG was also screened as a protective variable for aforementioned endpoints. Furthermore, the nested case–control study was used as an in-house validation for the prospective cohort study, which validated the results of cohort study. All of them demonstrated same conclusion that the definitive therapeutic effectiveness of HEG-TCM on the patients with CHD with revascularization is significantly different with the LEG and NEG.

Interestingly, the Cox regression models indicated that the simultaneously administration of clopidogrel and aspirin seemed to be a dangerous variable for the CHD endpoints. It is known that dual-antiplatelet therapies (DAPTs) are essential for thromboprophylaxis for the instable patients with CHD and PCI (30, 31). However, data are also emerging on long-term safety of antiplatelet agents and the bleeding hazards. Some studies indicated that a shorter period (≤6 months) of DAPT might have similar outcomes opposed to longer duration (>6 months) in patients undergoing PCI (21, 30, 31).

Of all the TCM used in the patients with CHD revascularization, Suxiaojiuxin pill and compound Danshen dropping pill accounted for substantial part. Based on TCM theories, Suxiaojiuxin pills have the function of blood-activating and qi-moving, meanwhile, compound Danshen dropping pill could activate the blood and resolve the thrombosis. The patients with CHD with the blood stasis syndrome (BSS) always manifest symptoms including precordial dull pain or stab pain, dark complexion, cyanotic nails and lips, scaly skin, purple dark tongue with spots or ecchymosis, and rough or knotted intermittent pulse (32, 33). In this study, the patients with CHD from three hospitals in Tianjin located in north of China were enrolled; the winter in Tianjin is much colder, i.e., a climate factor contributing to impede circulation of the Qi and blood (34), so that people in Tianjin are prone to be in Qi stagnation and BSS under the attacking of various pathogenies (35).

### Limitations

There were only 1,003 patients with CHD recruited from Tianjin, which might not very representative. The larger, multicenter cohort study throughout China is needed to reduce the recruitment bias. Second, the baseline characteristics (such as duration of CHD and smoking history) were limited to self-reported data and the recall bias was inevitable. We have checked for the Hospital Information System to remedy some recall bias of the data. Third, the confounding bias might also contribute to the evaluation of the effectiveness of TCM treatment on the CHD. The Cox regression models, the KM curves, and the nested case–control study were employed to minimize the unexpected bias. Fourth, we could not give detail descriptions of doses and formulations of TCM for all the participants because the TCM prescribed individually by physicians was not standardized, which yields to the theory of TCM. Fifth, in this study, the exact time after revascularization was not defined in the inclusion criteria. In fact, 80.27% patients were more than 1 year after revascularization showed that the situation of CHD disease was stable. Some subgroup analysis would be conducted further.

## Conclusion

The HEG-TCM may improve the outcomes of the patients with CHD after treatment of revascularization.

## Data Availability Statement

The original contributions presented in the study are included in the article/Supplementary Material, further inquiries can be directed to the corresponding author.

## Ethics Statement

This study involved human participants and was conducted in accordance with the recommendations of Ethics Committees of the Second Affiliated Hospital of Tianjin University of TCM and the protocol was approved by this ethics committees with number 2017-046-01. All subjects gave written informed consent from (ICF) in accordance with the Declaration of Helsinki. The patients/participants provided their written informed consent to participate in this study.

## Author Contributions

CL and HY wrote the manuscript. YZ, YL, JZ, SG, and RL designed the research. ZC, HD, YZ, YL, SG, and RL performed the research. CL, ZC, HD, and HY analyzed data. All authors contributed to the article and approved the submitted version.

## Funding

This study was supported by the National Natural Science Foundation of China (No. 81673751) and the National Major Science and Technology Projects of China (No. 2018ZX09734002).

## Conflict of Interest

The authors declare that the research was conducted in the absence of any commercial or financial relationships that could be construed as a potential conflict of interest.

## Publisher's Note

All claims expressed in this article are solely those of the authors and do not necessarily represent those of their affiliated organizations, or those of the publisher, the editors and the reviewers. Any product that may be evaluated in this article, or claim that may be made by its manufacturer, is not guaranteed or endorsed by the publisher.
