# Supplementary material for: Therapeutic Effects of Traditional Chinese Medicine for Patients With Coronary Heart Disease After Treatment of Revascularization: A Prospective Cohort Study in the Northern of China
Source: Front Cardiovasc Med. 2021 Nov 17;8:743262. doi: 10.3389/fcvm.2021.743262 (PMC8637924; doi:10.3389/fcvm.2021.743262)
Supplement: Supplementary file 1 [file Data_Sheet_1.PDF]

Supplemental Table I Baseline characteristics of the CHD patients (*n* = 1003)

Characteristics	*N* (%)
Age (years)	66.68±9.16
Gender	
Male	568 (56.63)
Female	435 (43.37)
CHD duration (years)	9.72±5.71
Drinking alcohol	
No	775 (77.27)
Yes	228 (22.73)
Smoking history	
No	536 (53.44)
Yes	467 (46.56)
Family history of CHD	
No	865 (86.24)
Yes	138 (13.76)
Heart rhythm	
Regluar	682 (68.00)
Arrhythmia（No AF）	238 (23.73)
AF	83 (8.27)
NYHA	
Ⅰ	54 (5.38)
Ⅱ	784 (78.17)
Ⅲ	133 (13.26)
Ⅳ	32 (3.19)
AMI	
No	671 (66.90)
Yes	332 (33.10)
Cerebral infarction	
No	793 (79.06)
Yes	210 (20.94)
AMI and cerebral infarction	
No	922 (91.92)
Yes	81 (8.08)
Diabetes	
No	620 (61.81)
Yes	383 (38.19)

Hyperlipidemia	
No	849 (84.65)
Yes	154 (15.35)
Hypertension	
No	211 (21.04)
Yes	792 (78.96)
Dual-antiplatelet (Clopidogrel + Aspirin)	
No	496 (49.45)
Yes	507 (50.55)
Anticoagulants	
No	967 (96.41)
Yes	36 (3.59)
Lipid-lowering agents	
No	174 (17.35)
Yes	829 (82.65)
Calcium channel blockers	
No	641 (63.91)
Yes	362 (36.09)
β-blockers	
No	361 (35.99)
Yes	642 (64.01)
ACEI＆ARB	
No	585 (58.33)
Yes	418 (41.67)
Nitrates	
No	353 (35.19)
Yes	650 (64.81)
TCM	
No	356 (35.49)
Yes	647 (64.51)

NEG is for non-exposure TCM group; LEG is for low-exposure TCM group; HEG is for high-exposure TCM group; CHD is for coronary heart disease; AF is for atrial fibrillation; NYHA is for New York Heart Association; AMI is for acute myocardial infarction ACEI is for angiotensin converting enzyme inhibitors; ARB is for Angiotensin receptor blocker; TCM is for Traditional Chinese Medicine
